# Identification of Hub Genes Correlated With Poor Prognosis for Patients With Uterine Corpus Endometrial Carcinoma by Integrated Bioinformatics Analysis and Experimental Validation

**DOI:** 10.3389/fonc.2021.766947

**Published:** 2021-11-19

**Authors:** Yi Yuan, Zhengzheng Chen, Xushan Cai, Shengxiang He, Dong Li, Weidong Zhao

**Affiliations:** ^1^ Department of Laboratory Medicine, Tongji Hospital, School of Medicine, Tongji University, Shanghai, China; ^2^ Department of Obstetrics and Gynecology, The First Affiliated Hospital of University of Science and Technology of China (USTC), Division of Life Sciences and Medicine, University of Science and Technology of China, Hefei, China; ^3^ Department of Clinical Laboratory, Maternal and Child Health Hospital of Jiading District, Shanghai, China; ^4^ School of Life Sciences and Technology, Tongji University, Shanghai, China

**Keywords:** uterine corpus endometrial carcinoma, weighted gene co-expression network analysis, protein-protein interaction network, hub gene, tumor differentiation

## Abstract

Uterine Corpus Endometrial Carcinoma (UCEC) is one of the most common malignancies of the female genital tract and there remains a major public health problem. Although significant progress has been made in explaining the progression of UCEC, it is still warranted that molecular mechanisms underlying the tumorigenesis of UCEC are to be elucidated. The aim of the current study was to investigate key modules and hub genes related to UCEC pathogenesis, and to explore potential biomarkers and therapeutic targets for UCEC. The RNA-seq dataset and corresponding clinical information for UCEC patients were obtained from the Cancer Genome Atlas (TCGA) database. Differentially expressed genes (DEGs) were screened between 23 paired UCEC tissues and adjacent non-cancerous tissues. Subsequently, the co-expression network of DEGs was determined *via* weighted gene co-expression network analysis (WGCNA). The Blue and Brown modules were identified to be significantly positively associated with neoplasm histologic grade. The highly connected genes of the two modules were then investigated as potential key factors related to tumor differentiation. Additionally, a protein-protein interaction (PPI) network for all genes in the two modules was constructed to obtain key modules and nodes. 10 genes were identified by both WGCNA and PPI analyses, and it was shown by Kaplan-Meier curve analysis that 6 out of the 10 genes were significantly negatively related to the 5-year overall survival (OS) in patients (AURKA, BUB1, CDCA8, DLGAP5, KIF2C, TPX2). Besides, according to the DEGs from the two modules, lncRNA-miRNA-mRNA and lncRNA-TF-mRNA networks were constructed to explore the molecular mechanism of UCEC-related lncRNAs. 3 lncRNAs were identified as being significantly negatively related to the 5-year OS (AC015849.16, DUXAP8 and DGCR5), with higher expression in UCEC tissues compared to non-tumor tissues. Finally, quantitative Real-time PCR was applied to validate the expression patterns of hub genes. Cell proliferation and colony formation assays, as well as cell cycle distribution and apoptosis analysis, were performed to test the effects of representative hub genes. Altogether, this study not only promotes our understanding of the molecular mechanisms for the pathogenesis of UCEC but also identifies several promising biomarkers in UCEC development, providing potential therapeutic targets for UCEC.

## Introduction

Uterine corpus endometrial carcinoma (UCEC) is one of the leading causes of cancer-associated mortality in women worldwide. It is reported by the GLOBOCAN 2020 of the International Agency for Research on Cancer that the global incidence and mortality age-standardized rates (ASRs) for UCEC are 8.7 per 100,000 women and 1.8 per 100,000 women respectively (1). Increase in both rates of incidence and associated mortality together has made UCEC an important consideration for women’s health. Despite great advances have been made regarding to the diagnosis and treatment of UCEC, prognosis of the disease remains unsatisfactory, especially for advanced UCEC. While many early UCEC patients usually have been received with good prognosis, poor clinical outcomes still commonly exist in significant numbers of women with more aggressive variants of UCEC (2, 3).

As the application of next generation sequencing (NGS) infiltrates into clinical studies, a greater volume of genomic data and patient information has become available online and our understanding of molecular biology has been substantially improved (4, 5). Therefore, the molecular diversity and genetic heterogeneity of UCEC has been revealed by growing evidence, and the integration of biologically relevant molecular information into the UCEC diagnoses has provided molecular subclass-specific treatment stratification with opportunities (6, 7). For example, four distinct molecular subtypes of UCEC, carrying significant prognostic and predictive information, have been identified by TCGA through an integrated genomic analysis (8). Embracing and incorporating these subtypes into clinical practice is thus attractive. However, it is too expensive and cumbersome of the method applied by TCGA to be widely implemented in routine clinical practice. Besides, only a few studies have conducted a comprehensive analysis of DEGs related to risk judgment and prognosis of UCEC (9, 10), and hence more work is needed to implement biological rationale for targeted therapies and improve outcomes for UCEC patients.

The present study aimed to identify potential key molecules with prognostic significance by bioinformatic methods and to improve our understanding of the genetic landscape of UCEC. We first screened DEGs (including mRNAs and lncRNAs) based on the TCGA gene expression data of paired UCEC tissues and adjacent non-tumor endometrial tissues. WGCNA algorithm was then applied to construct the co-expression network of DEGs in UCEC. Combined with the analysis of PPI network, highly correlated gene modules and key genes that were mostly related to the clinical traits of UCEC were found. In addition, the lncRNA-miRNA-mRNA and lncRNA-transcription factor (TF)-mRNA networks were constructed to explore the molecular mechanisms of UCEC-related lncRNAs. After a range of screening, 6 mRNA strands and 3 lncRNA strands with prognostic predictive potential were identified, which could significantly distinguish well-differentiated UCEC (neoplasm histologic grade G1-G2) from poorly differentiated UCEC (neoplasm histologic grade G3) and be negatively related to prolonged patient survival time. Finally, we experimentally validated their expression patterns in UCEC cells, and AURKA and DUXAP8 were selected as representative hub genes for functional verification.

## Materials and Methods

### Data Collection and Processing

RNA-seq expression data from a total of 548 patients with UCEC and corresponding clinical information were obtained from the TCGA database (https://portal.gdc.cancer.gov/projects/TCGA-UCEC). LncRNAs were annotated by human gene annotation files (GRCh38.p12), which were downloaded from the Ensembl database (https://asia.ensembl.org/index.html). We excluded UCEC patients from TCGA dataset according to the following criteria: 1) patients with incomplete clinical information; 2) patients with overall survival (OS) time or follow-up time less than 30 days. Finally, 524 UCEC patients were selected in our study.

### Differential Gene Expression and Principal Component Analysis

Among the tissues of these patients, 23 paired UCEC tissues and adjacent non-cancerous tissues were selected to screen out DEGs by using the edgeR package (version 3.32.1) (11). 1) Filter the data to remove genes with low counts. Genes that have count-per-million (CPM) values above one in at least two samples were kept; 2) Normalize the data. Trimmed Mean of M-values (TMM) normalization method was used for the data analysis; 3) Explore the data and estimate the dispersion; 4) Investigate DEGs. The false discovery rate (FDR) was applied for multiple testing correction of raw *P* values through the Benjamini-Hochberg method. A |log2 fold change (FC)| > 2 and an FDR < 0.05 were set as the threshold for identifying DEGs. Besides, the samples were clustered based on gene expression data by performing principal component analysis (PCA). Differentially expressed mRNAs (DEmRNAs) and lncRNAs (DElncRNAs) were used for further analysis.

### Construction of Weighted Co-Expression Network and Gene Function Enrichment of the Clinically Significant Modules

WGCNA was used to construct the co-expression network of DElncRNAs and DEmRNAs in UCEC according to the following main steps: 1) Select the weighting coefficient, β; 2) Transform the gene expression profiles into an adjacency matrix; 3) Use the adjacency matrix to define a separate measure of similarity, the Topological Overlap Matrix(TOM); 4) Perform hierarchical clustering for TOM-based dissimilarity (dissTOM) to obtain the hierarchical clustering tree; 5) Identify the modules *via* dynamic branch cutting methods; 6) Calculate the module eigengene (ME) of each module; 7) Identify the modules that were most strongly related to the clinical traits (12, 13). Next, gene ontology (GO) annotation and Kyoto encyclopedia of genes and genomes (KEGG) pathway enrichment were performed using the clusterProfiler R package (version 3.18.1) (14). Significant enriched functions and pathways were visualized by ggplot2 R package (version 3.3.5).

### Identification of Clinically Significant Hub Genes

Two methods were applied to screen the hub genes of the network. In the first method, the network screening function based on GS (representing the correlation between the gene and a given clinical trait) and MM (representing the correlation between the gene and a given module) was used to directly identify hub genes. A cut-off criteria (|MM| > 0.8 and |GS| > 0.4) was employed to obtain key genes with high connectivity in the clinically significant modules. In the second method, a PPI network was built based on the online STRING database (version 11.5) and analyzed using Cytoscape (version 3.8.0) (15). Common genes that scored in the top 30 by all five methods in CytoHubba were selected as key nodes of UCEC, and MCODE in Cytoscape was used to perform module analysis (16, 17). Overlapping genes identified by both WGCNA and PPI analyses were designated as potential hub genes for further validation and analysis (18, 19).

### Gene Set Enrichment Analysis for Hub Genes

Based on the median expression value of the hub genes, samples of TCGA UCEC were divided into low- and high-expression phenotypes. Then, GSEA (version 1.52.1) was performed to detect which KEGG pathways were enriched (20). Terms with FDR < 0.05 were visualized by ggplot2 R package to investigate potential functions of the hub genes.

### Construction of the PPI, lncRNA-miRNA-mRNA ceRNA, and lncRNA-TF-mRNA Networks

The lncRNA-miRNA-mRNA ceRNA and lncRNA-TF-mRNA networks in the co-expression modules were constructed based on several online databases (Starbase, miRWalk, LncTarD, RNA Interactome and LncMAP) and visualized in Cytoscape (version 3.8.0) (21–25).

### Survival Analysis

To identify prognostic hub genes by integrating the clinical information of patients suffering from UCEC in TCGA, Kaplan-Meier curve analysis of the samples with hub genes was conducted with the survival R package (version 3.2-10), and significant differences in survival were determined with the log-rank test. *P* values <0.05 were regarded as significant.

### Cell Lines and Cell Culture

The human UCEC cell lines Ishikawa and KLE were purchased from Procell Life Science &Technology Co., Ltd. and cultured in Dulbecco’s modified Eagle’s medium (DMEM) and DMEM/F12 medium respectively, supplemented with 10% fetal bovine serum and 1% penicillin/streptomycin and maintained with 5% CO_2_ at 37°C in a humidified incubator.

### RNA Extraction and qRT-PCR

Total cellular RNAs were extracted from Ishikawa and KLE by using TRIzol™ Reagent (Thermo Fisher Scientific Inc., United States) according to the manufacturer’s protocol. cDNA was synthesized using MMLV Reverse Transcriptase (Anhui Toneker Biotechnology Co., Ltd., China). The qRT-PCR analysis on mRNA and lncRNA was performed using 2 × PCR SYBR Green Mix buffer (Anhui Toneker Biotechnology Co., Ltd., China). The PCR process ran 40 cycles for 15s at 95°C and for 1 min at 60°C in Applied Biosystems 7500 Real-Time PCR System (Thermo Fisher Scientific Inc., United States). The 2[-Delta Delta C(T)] was adopted to calculate relative expression with GAPDH as an internal control (26). The primers used were listed in **Table 1**.

**Table 1:** The primers for select genes.

Gene	Forward primer	Reverse primer
AURKA	CAGTACATGCTCCATCTTCCAG	AAAGAACTCCAAGGCTCCAG
BUB1	GCTGGCTTGGCACTGATTGA	GACCTTCAGGCTTACACTCTCC
CDCA8	CAGCAGCAGGAGAGCGGATT	ATTTGGGCGAGACGGTTGGA
DLGAP5	TCTTATTCGCACAGCAGTTGGT	GCCACCCAGATTCCTCAAGTTT
KIF2C	CCAAGGAAGAGGAGGAACTGTC	ATAGTCTGGCTGCTCGGTCAT
TPX2	GCTGGAGAAGAGAATGGCTGAG	GCAGTGGAATCGAGTGGAGAAT
AC015849.16	CAGCTCAGCTCTCCTCAGACAT	CTGCTGGACTGGAGAAGGTTCA
DGCR5	CTGGAGATGGAGAAGCGAACC	GGAGACACAGACCACAAGAGAC
DUXAP8	CACTGATTCCTTCTGAGACT	GAGCCATACTGTTGAACCT

### Plasmid Construction and Stable Transfection

Three oligonucleotides specific for short hairpin RNAs (shRNAs) against DUXAP8 were inserted into the lentiviral expression vector (pLVX-shRNA1) respectively. Lentiviral vectors were then cotransfected with packaging plasmids psPAX2 and pMD2G into HEK-293T cells. The viral supernatant was harvested 48 and 72 h after transfection, filtered through a 0.45 μm millipore filter, aliquoted and stored at -80°C for subsequent use. Stable cell lines were obtained by selection with puromycin. The interference sequences targeting DUXAP8 were listed in **Table 2**.

**Table 2:** The interference sequences targeting DUXAP8.

shRNA sequences	
sh-DUXAP8 1#	GGAACTTCCCAAACCTCCATGATTT
sh-DUXAP8 2#	AAGATAAAGGTGGTTTCCACAAGAA
sh-DUXAP8 3#	CAGCATACTTCAAATTCACAGCAAA

### CCK-8 Assays

Cells were seeded in 96-well plates at a density of 4×10^3^ cells/well in triplicate and incubated at 37°C. Cell viability was determined by a Cell Counting Kit-8 (CCK-8) kit (Dojindo Laboratories, Japan) according to the manufacturer’s instructions. The absorbance at 450 nm was measured after incubation at 37°C for 2 h with microtiter plate reader (BioTek, United States), and cell viability was calculated.

### AURKA Inhibitor Treatment and Cell Viability

Alisertib (MLN8237) was purchased from Selleck, dissolved in dimethyl sulfoxide (DMSO) and kept frozen at -20°C. Cells were dispensed into 96-well plates at a density of 4×10^3^ cells in 100 μL of complete medium with different concentrations of Alisertib (0.01, 0.1, 1, 10, 50, 100 μM) for 72 h. The final concentration of DMSO in medium was kept at 0.2% for all groups including control samples (no Alisertib, DMSO only). CCK-8 assays were then performed to detect cell viability.

### Cell Clonogenic Assay

Cells were seeded into 6-well plates at a density of 5×10^2^ cells, followed by incubation at 37 °C for 1-3 weeks. Then, colonies were fixed with glutaraldehyde (6.0% v/v) and stained with crystal violet (0.5% w/v). The colony was defined to consist of at least 50 cells and colonies were quantified by using ImageJ image analysis software (27).

### Cell Cycle Distribution and Apoptosis Analysis

Cells were seeded at a density of 3×10^5^ cells/mL and treated with the Alisertib (1μM) for 48 h. For cell cycle distribution analysis, cells were harvested and fixed with 70% cold ethanol at -20°C overnight. The fixed cells were then resuspended and stained with propidium iodide (PI)/RNase staining buffer (BD Biosciences, United States) according to the manufacturer’s instructions. The cells were analyzed by flow cytometry (BD Biosciences, United States) and the proportions of cells at each phase of the cell cycle were determined and compared using the ModFit LT software. For cell apoptosis analysis, cells were harvested and stained with Annexin V-FITC/PI apoptosis kit (MULTISCIENCES, China) following the manufacturer’s instructions and the data were analyzed with Flowjo software. Annexin V^+^/PI^–^ cells were considered in early apoptosis, Annexin V^+^/PI^+^ populations denoted late apoptotic/necrotic cells (28).

### Statistical Analysis

Statistical analyses are based on RStudio software (version 3.6.3) and GraphPad Prism (version 8.0). The experimental data are presented as the means ± standard deviation (SD). The independent Student’s t-test was used to compare equivalent variables with normal distribution between two groups. Statistical differences between groups with unequal sample sizes and different variances were assessed using unpaired t-tests with Welch correction (Welch t-tests) (29). Multiple group comparisons were performed with one-way analysis of variance (ANOVA) followed by Tukey’s multiple comparison test. *P* < 0.05 was considered statistically significant. All data generated or analyzed during the current study are included in the published article and **Supplementary Materials**.

## Results

### Identification of Differentially Expressed lncRNAs and mRNAs

**Figure 1:**
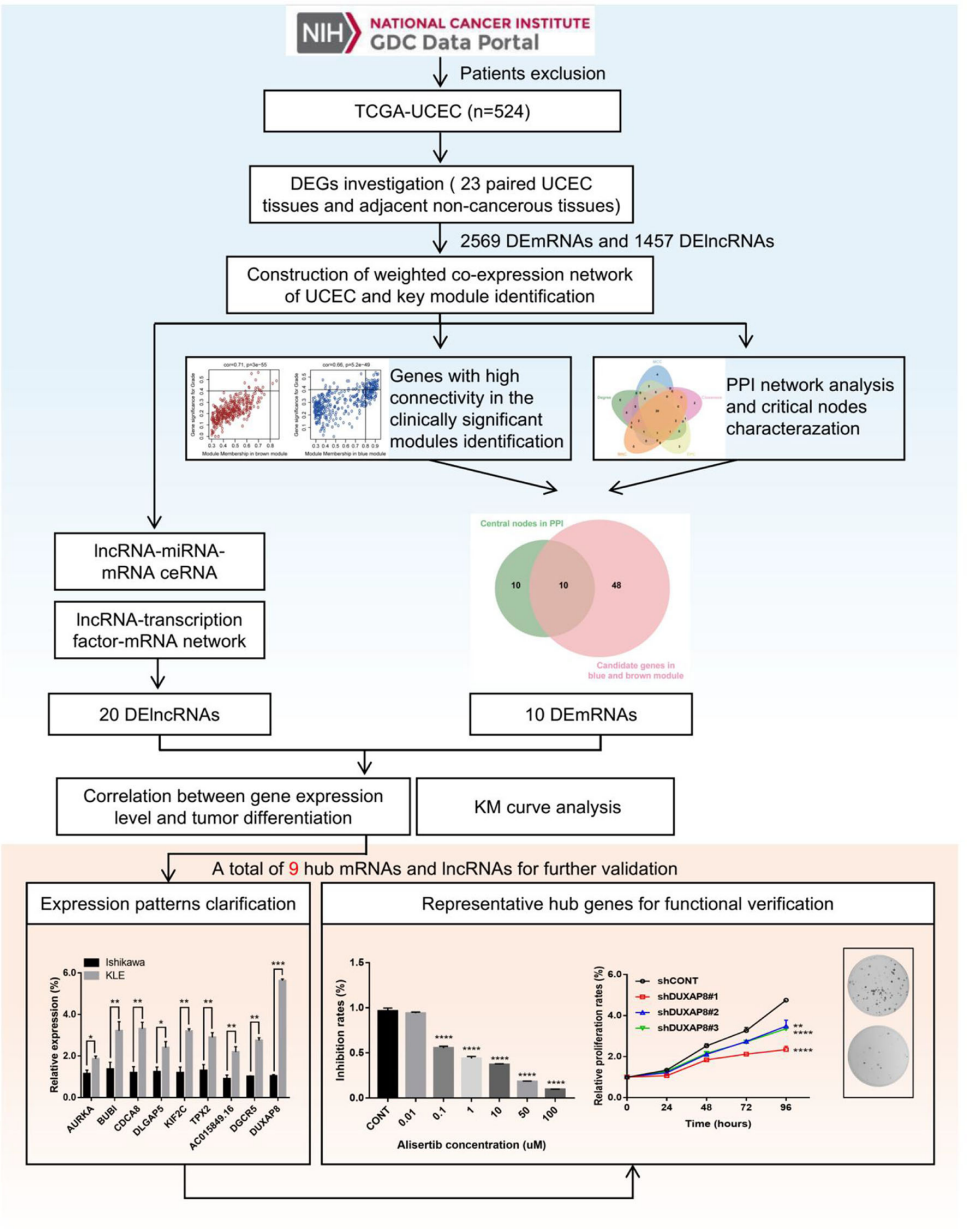
Flowchart of the study. 548 patients with UCEC and corresponding clinical information were obtained from the TCGA database. According to the exclusion criteria, a total of 24 cases were excluded, and 524 UCEC patients were selected in this study. DEGs (including mRNAs and lncRNAs) were screened based on the gene expression data of 23 paired UCEC tissues and adjacent non-tumor endometrial tissues. Further, WGCNA algorithm was applied to construct the co-expression network of DEGs. Combined with PPI network analysis, highly correlated gene modules and key genes that were mostly associated with the clinical traits of UCEC were identified. Besides, the lncRNA-miRNA-mRNA and lncRNA-TF-mRNA networks were constructed to investigate the molecular mechanisms of UCEC-related lncRNAs. Finally, after a range of screening, 6 mRNA strands and 3 lncRNA strands with prognostic predictive potential were identified, of which expression patterns were experimentally validated. Moreover, AURKA and DUXAP8 were selected as representative hub genes for functional verification.

**Figure 2:**
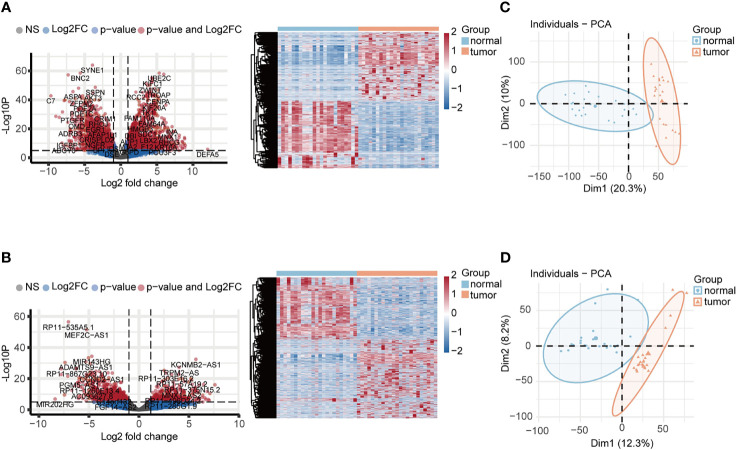
Identification of DEGs associated with UCEC. **(A, B)** The volcano plots and heatmaps of DEmRNAs and DElncRNAs. 2569 DEmRNA strands (1295 upregulated and 1274 downregulated) and 1457 DElncRNA strands (733 upregulated and 724 downregulated) were identified at the threshold of FDR < 0.05 and |log2FC| > 2. **(C, D)** PCA plots of DEmRNAs and DElncRNAs showed that 23 tumors were clustered separately from their paired adjacent non-cancerous tissues.

The schematic overview of this study was shown in **Figure 1**. The RNA-seq expression dataset and corresponding clinical information for patients with UCEC were obtained from the TCGA UCEC cohort. Once the raw data was normalized, DEG analysis was performed between 23 paired UCEC tissues and adjacent non-cancerous tissues. In total, 2569 DEmRNA strands (1295 upregulated and 1274 downregulated) and 1457 DElncRNA strands (733 upregulated and 724 downregulated) were identified at the threshold of FDR < 0.05 and |log2FC| > 2 and selected for further analysis. Volcano plots and heatmaps were plotted to show the distribution of DEmRNAs (**Figure 2A**) and DElncRNAs (**Figure 2B**) that were differentially expressed in UCEC tissues in comparison to nontumorous tissues. It was also demonstrated by PCA that the 23 tumors were clustered separately from their paired adjacent non-cancerous tissues (**Figures 2C, D**).

### Construction of Weighted Co-Expression Network of UCEC and Key Module Identification

**Figure 3:**
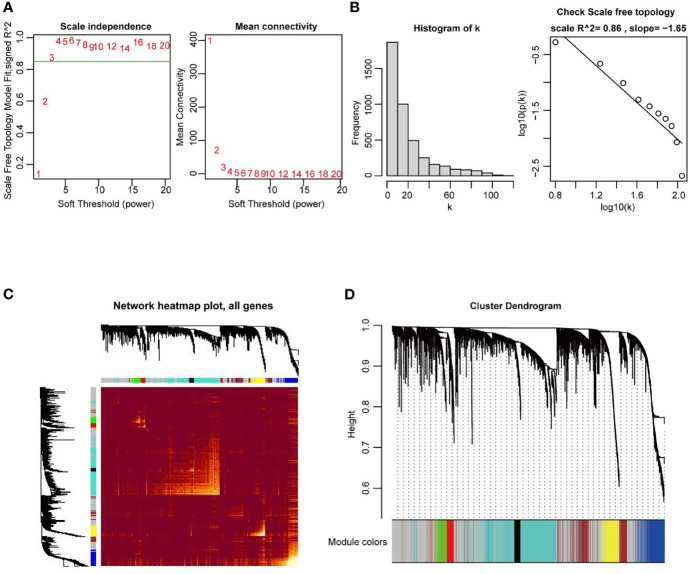
Construction of weighted co-expression network of DEGs in UCEC. **(A, B)** Examination of the scale−free fit index for distinct soft-thresholding powers (β), and average network connectivity under different weighting coefficients. β = 3 was selected as a soft threshold to construct the co-expression modules. **(C)** Heatmap depicting the TOM among DEGs based on the co-expression modules. **(D)** 8 modules were characterized due to a dissimilarity measure. Within the 8 modules, the grey module was a gene set in which the genes were not clustered into any modules.

In the next stage, the co-expression network of all DEGs in UCEC, including 2569 DEmRNA strands and 1647 DElncRNA strands, was constructed by using the WGCNA R package. When the weighting coefficient β was 3, the independence degree was > 0.8, indicating that the scale free topology of the network could be appropriately assessed by the power value according to the WGCNA algorithm (**Figures 3A, B**). Therefore, β = 3 was selected as a soft threshold to construct the co-expression modules. The gene expression profiles were transformed into the adjacency matrix, and dissTOM for DEGs was then obtained (**Figure 3C**). 8 modules were detected by the method of dynamic tree cutting and merging similar modules (**Figure 3D**).

**Figure 4:**
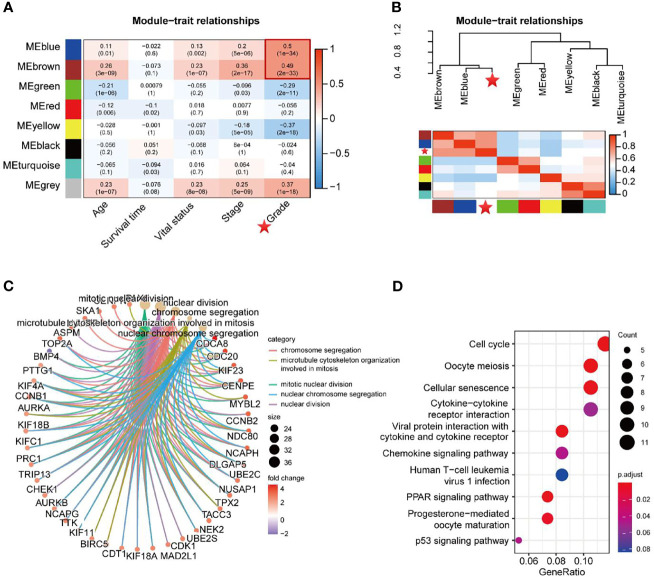
Identification of key modules related to the clinical traits. **(A)** Module−trait heatmap of the correlation between module eigengenes and 5 clinical traits of UCEC. The *P*-values of each module’s correlation with the corresponding clinical trait were shown in parentheses. **(B)** Hierarchical clustering dendrogram of MEs and neoplasm histologic grade (labeled by asterisk). Heatmap of the adjacencies in the eigengene network, including neoplasm histologic grade. **(C, D)** GO term enrichment and KEGG pathway analysis for DEGs in the Blue and Brown modules.

The Pearson correlation coefficients for the MEs of all modules and the clinical information were calculated to identify which modules were related to the clinical traits. As shown in **Figure 4A**, the Blue module (R = 0.50, *P* = 1e-34) and Brown module (R = 0.49, *P* = 2e-33) were significantly positively associated with neoplasm histologic grade. Moreover, groups of correlated eigengenes were identified through hierarchical clustering dendrogram and heatmap construction, which indicated that the Blue and Brown modules were most significantly associated with neoplasm histologic grade (**Figure 4B**), suggesting that genes in the two modules might have potential roles in tumor differentiation and progression. Therefore, the two modules mentioned above were selected as the clinically significant module for further analysis. GO term enrichment analysis demonstrated that genes in the two modules were significantly enriched in the following biological processes which are ‘Mitotic nuclear division’, ‘Nuclear division’, ‘Chromosome segregation’, ‘Microtubule cytoskeleton organization involved in mitosis’ and ‘Nuclear chromosome segregation’ (**Figure 4C** and **Table S1**). In the KEGG pathway enrichment analysis, the top 10 enriched pathways were ‘Cell cycle’, ‘Oocyte meiosis’, ‘cellular senescence’, ‘Cytokine-cytokine receptor interaction’, ‘Viral protein interaction with cytokine and cytokine receptor’, ‘Chemokine signaling pathway’, ‘Human T-cell leukemia virus 1 infection’, ‘PPAR signaling pathway’, ‘Progesterone-mediated oocyte maturation’ and ‘P53 signaling pathway’ (**Figure 4D** and **Table S2**).

### Identification of Hub Genes

**Figure 5:**
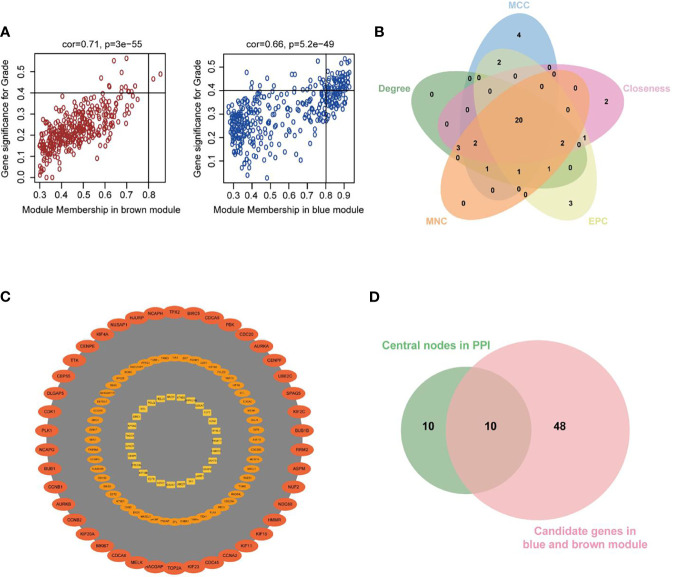
Investigation of hub genes in the Blue and Brown modules. **(A)** Scatterplots of GS for neoplasm histologic grade (y-axis) *versus* MM (x-axis) in the two modules. **(B)** Key nodes were analyzed by CytoHubba using the following five methods: Closeness, Degree, edge percolated component (EPC), Maximum neighborhood component (MCC), and Maximum Neighborhood Component (MNC). **(C)** The most significant module obtained from the PPI network (MCODE score = 105.433). **(D)** Venn diagram demonstrated overlapping genes of the WGCNA and PPI network.

The highly connected genes of the Blue and Brown modules were investigated as potential key factors related to tumor differentiation. Based on the cut-off criteria (|MM| > 0.8 and |GS| > 0.4), 58 genes with high connectivity in the clinically significant modules were identified (30, 31) (**Figure 5A**). Additionally, we also constructed a PPI network for all genes in the two modules based on STRING database and Cytoscape software, which was composed of 532 nodes and 9911 edges. 20 common genes that scored in the top 30 by all five methods in CytoHubba were selected as key nodes of UCEC in PPI analysis (**Figure 5B**). MCODE in Cytoscape was used to perform module analysis. We found that all of the 20 common genes were in module 1, which was the fairly significant module (MCODE score = 105.433) in all modules and potentially played an important role in UCEC progression (**Figure 5C** and **Table S3**).

**Figure 6:**
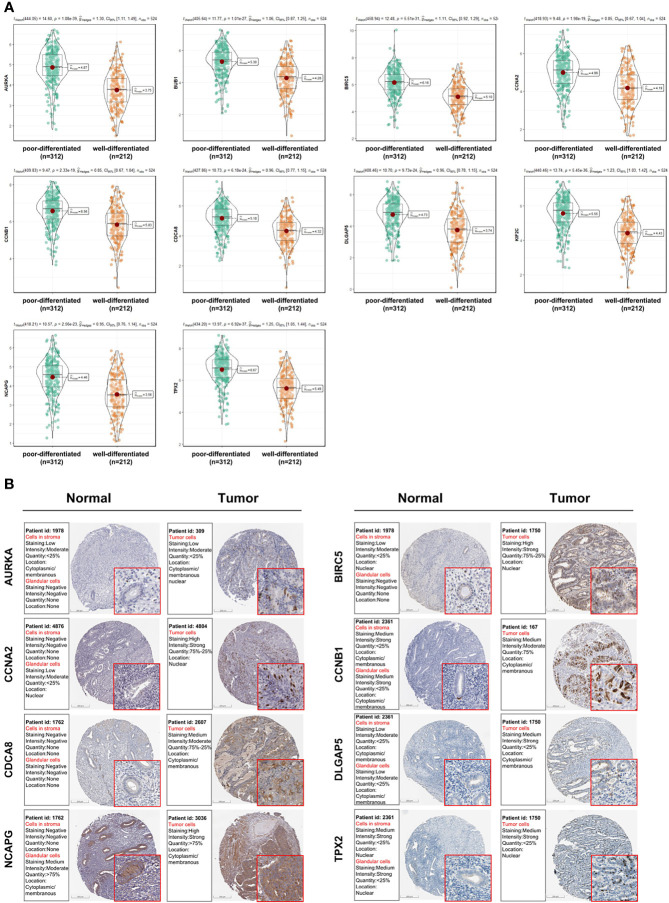
Analysis of the expression patterns of candidate genes (AURKA, BIRC5, BUB1, CCNA2, CCNB1, CDCA8, DLGAP5, KIF2C, NCAPG and TPX2). **(A)** The expression levels of candidate genes between poorly and well-differentiated UCEC in the TCGA dataset. **(B)** The protein expressions of candidate genes between UCEC tissues and non-tumorous tissues based on the Human Protein Atlas database. Most of these genes were higher in UCEC tissues compared to non-tumorous tissues, except that no protein expression was detected for KIF2C gene and there was no expression data for BUB1 gene in this database.

**Figure 7:**
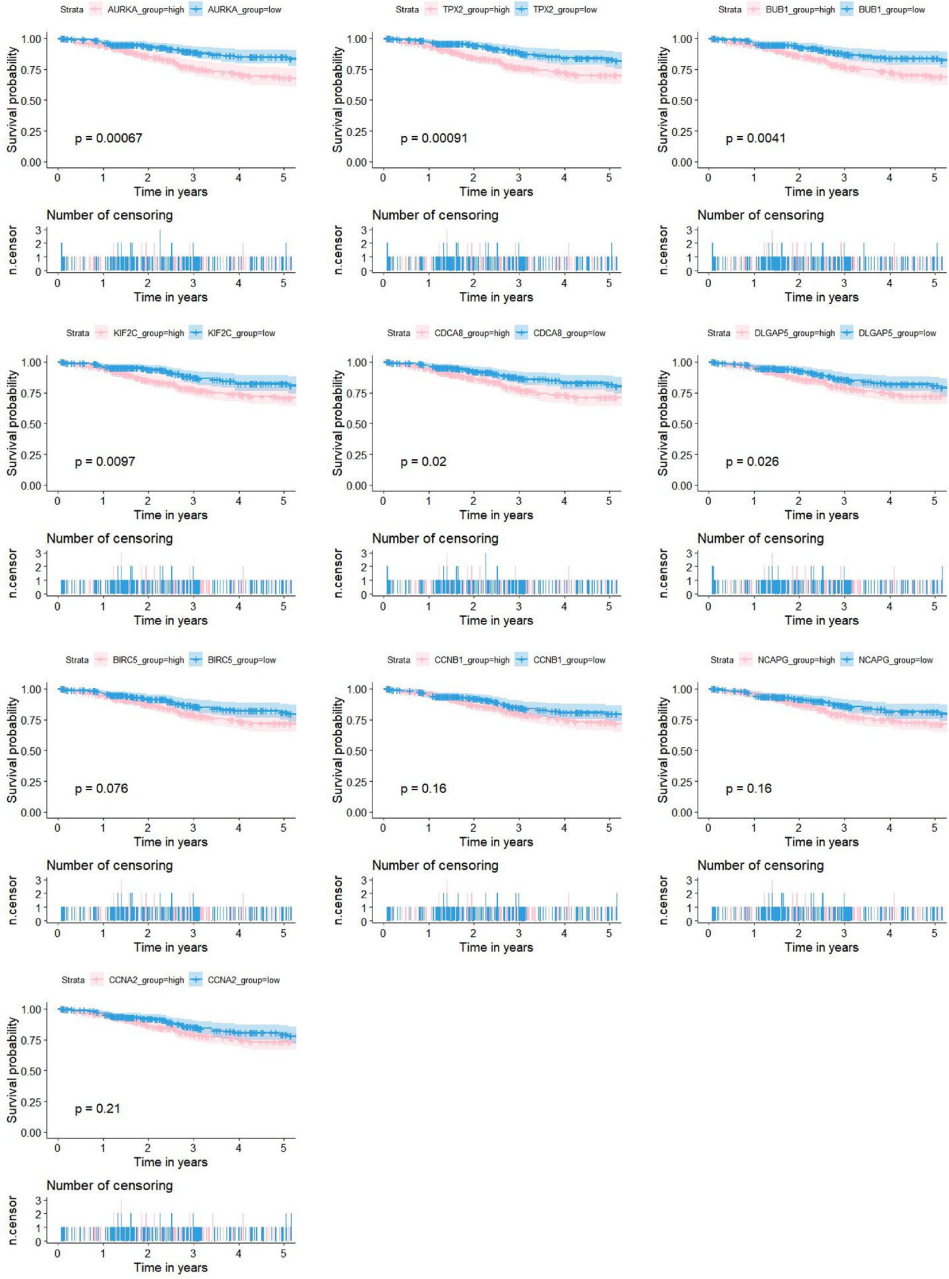
Associations between the expression levels of candidate genes (AURKA, BIRC5, BUB1, CCNA2, CCNB1, CDCA8, DLGAP5, KIF2C, NCAPG and TPX2) and the 5-year OS for 524 patients with UCEC based on the TCGA dataset. 6 of the 10 genes were significantly negatively related to prolonged patient survival time (AURKA, BUB1, CDCA8, DLGAP5, KIF2C and TPX2).

**Figure 8:**
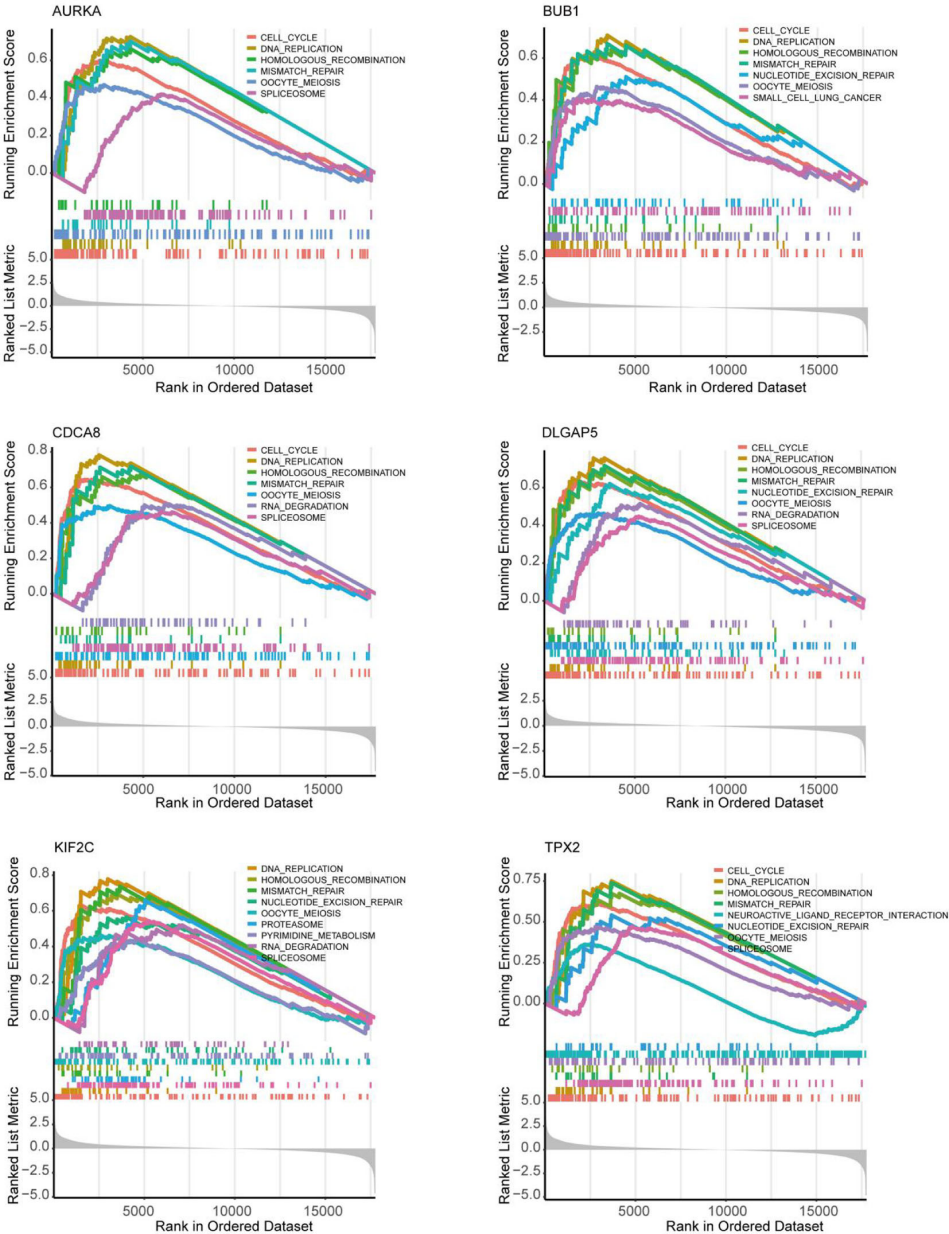
Exploration of the possible pathogenesis of 6 hub genes (AURKA, BUB1, CDCA8, DLGAP5, KIF2C and TPX2) in UCEC by using the GSEA algorithm. 5 common functional gene sets, ‘Cell cycle’, ‘DNA replication’, ‘Mismatch repair’, ‘Homologous recombination’ and ‘Oocyte meiosis’, were significantly enriched in UCEC samples with high gene expression.

As shown in **Figure 5D**, 10 genes identified by both WGCNA and PPI analyses were designated as potential candidates for further validation and analysis (AURKA, BIRC5, BUB1, CCNA2, CCNB1, CDCA8, DLGAP5, KIF2C, NCAPG and TPX2). Based on the TCGA data, the expression levels of the 10 genes could be used to significantly distinguish well-differentiated UCEC (neoplasm histologic grade G1-G2) from poorly differentiated UCEC (neoplasm histologic grade G3) (**Figure 6A**) (*P* < 0.0001). The protein expressions of most of these genes were higher in UCEC tissues compared to non-tumor tissues, according to the Human Protein Atlas database (32) (**Figure 6B**), except that no protein expression was detected for KIF2C gene and there was no expression data for BUB1 gene in this database. Moreover, Kaplan-Meier curve analysis showed that 6 of the 10 genes were significantly negatively related to the 5-year overall survival (OS) of patients with UCEC (AURKA, BUB1, CDCA8, DLGAP5, KIF2C and TPX2) (**Figure 7**). Therefore, these 6 genes were selected as representative genes for further investigation. Then, GSEA was performed to explore the possible pathogenesis of the 6 genes in UCEC. Five common gene sets, ‘Cell cycle’, ‘DNA replication’, ‘Mismatch repair’, ‘Homologous recombination’ and ‘Oocyte meiosis’, were enriched in the sample group with high gene expression (FDR < 0.05; **Figure 8**). Overall, these gene sets were tightly associated with cell proliferation, indicating a vital role of the 6 genes in regulating the cell cycle and proliferation of UCEC.

### Construction of lncRNA-miRNA-mRNA ceRNA and lncRNA-Transcription Factor (TF)-mRNA Networks

**Figure 9:**
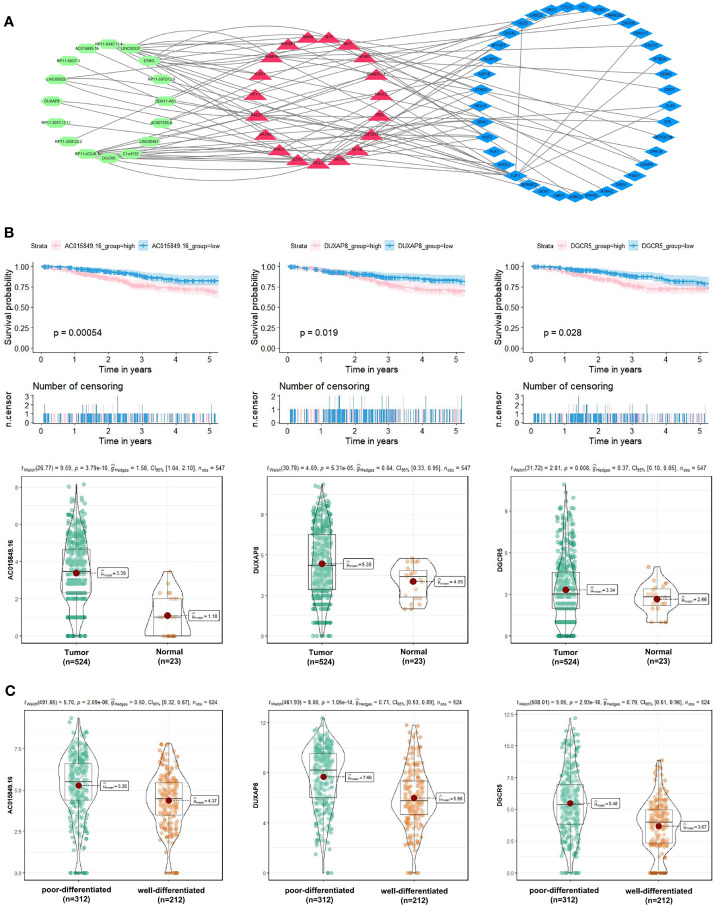
Exploration of the molecular mechanism of UCEC-related lncRNA in the Blue and Brown modules. **(A)** Construction of lncRNA-transcription factor (TF)-mRNA networks. **(B)** 3 lncRNA strands (AC015849.16, DUXAP8 and DGCR5) were significantly negatively related to the 5-year OS of patients with UCEC, with higher expression levels in UCEC tissues compared to non-tumorous tissues. **(C)** The expression levels of AC015849.16, DUXAP8 and DGCR5 between poorly and well-differentiated UCEC tissues in the TCGA dataset.

Besides, to explore the molecular mechanism of UCEC-related lncRNA, lncRNA-miRNA-mRNA and lncRNA-TF-mRNA networks were constructed from several online databases (Starbase, miRWalk, LncTarD, RNA Interactome and LncMAP), according to the DElncRNAs and DEmRNAs from the co-expression Blue and Brown modules. The lncRNA-miRNA-mRNA ceRNA networks consisted of 4 DElncRNA strands, 4 DEmiRNA strands, and 8 DEmRNA strands (**Table S4**). The lncRNA-TF-mRNA networks consisted of 16 DElncRNA strands, 37 DEmRNA strands, and 20 transcription factors (**Figure 9A** and **Table S5**). 3 lncRNA strands in the constructed networks were identified as being significantly associated with decreased 5-year OS (AC015849.16, DUXAP8 and DGCR5), with higher expression levels in UCEC tissues compared to non-tumorous tissues (**Figure 9B**). Moreover, the expressions of AC015849.16, DUXAP8 and DGCR5 were significantly upregulated in poorly differentiated UCEC tissues compared with well-differentiated UCEC tissues (**Figure 9C**) (*P* < 0.0001).

### Expression and Function Analysis of the Selected Hub mRNAs and lncRNAs *In Vitro*

**Figure 10:**
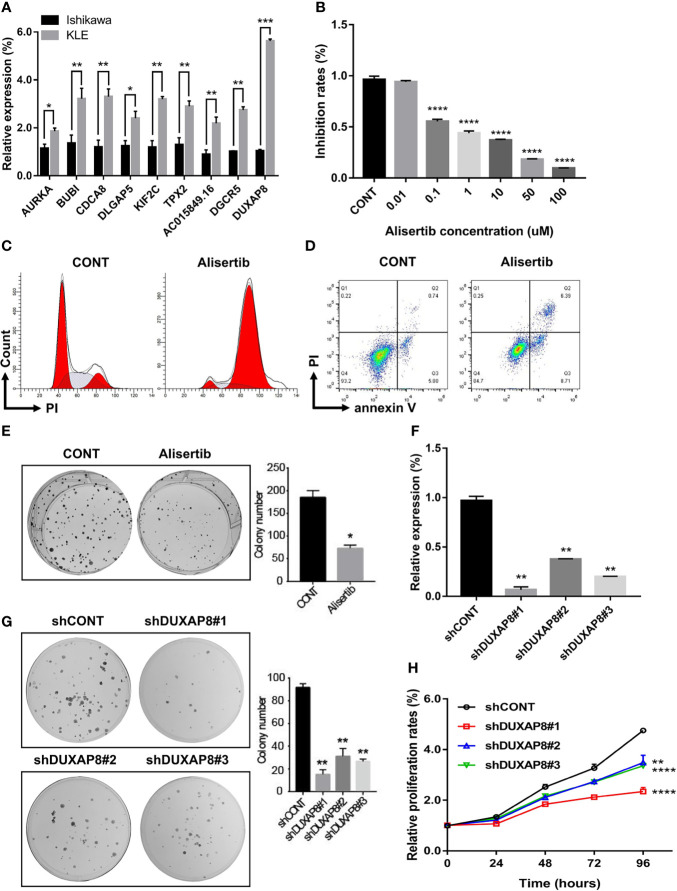
Expression and functional validation of the selected hub mRNAs and lncRNAs *in vitro*. **(A)** The expression patterns of these genes in two endometrial cancer cell lines, including Ishikawa (histological grade 1; G1) and KLE (histological grade 3; G3). **(B–E)** The biological impacts of suppressing AURKA with Alisertib (MLN8237) in UCEC cells *in vitro*. Alisertib decreased Ishikawa cells viability in a dose-dependent manner, induced G2/M phase arrest and enhanced cellular apoptosis. Furthermore, Alisertib limited the long-term clonogenic survival (1 μM). **(F)** DUXAP8-shRNA/GFP lenti-virus (*versus* control virus) was used to knock down DUXAP8. **(G, H)** Downregulation of DUXAP8 inhibited the colony formation and impaired cells growth. Data are the mean ± SD of three independent experiments. *P < 0.05, **P < 0.01, ***P < 0.001, ****P < 0.0001.

To confirm the reliability of the hub genes from WGCNA and PPI, lncRNA-miRNA-mRNA ceRNA, and lncRNA-TF-mRNA network, we verified the expression patterns of these genes in two endometrial cancer cell lines, including Ishikawa (histological grade 1; G1) and KLE (histological grade 3; G3) (33). All of these hub genes presented higher expression in KLE than in Ishikawa cells (**Figure 10A**), which were consistent with the results obtained for the TCGA UCEC cohort.

Among these hub genes, AURKA was the most significant gene associated with the survival rate in UCEC patients. Therefore, AURKA was selected as a representative gene for further verification. We evaluated the biological impact of inhibiting AURKA with Alisertib (MLN8237), an investigational, oral, and selective inhibitor of AURKA in UCEC cells *in vitro*. The cell viability (3 days), in response to Alisertib treatment, was determined by CCK-8 assay. It was discovered that Alisertib decreased cell viability in a dose-dependent manner (**Figure 10B**). To further assess the effects of AURKA on cell cycle distribution, Ishikawa cells were treated with Alisertib (1 μM) for 24h and the DNA content was then measured by flow cytometry. As shown in **Figure 10C**, incubation of cells with Alisertib resulted in a marked G2/M phase arrest from 14.0% at basal level to 84.6%. Moreover, treatment with Alisertib also increased the total proportion of apoptotic cells (early + late apoptosis). Compared to the control group (0.01% DMSO), there was 2.3-fold increase in total apoptotic cells when Ishikawa cells were incubated with Alisertib (1 μM) (**Figure 10D**). In the long-term clonogenic survival assay, after a single overnight treatment with Alisertib (1 μM), a significant reduction in the number of viable cell colonies in UCEC cells was demonstrated (**Figure 10E**). Collectively, these results indicate that inhibition of AURKA using Alisertib can effectively suppress cancer cell viability, modulate cell cycle distribution and induce apoptosis in UCEC.

We also selected DUXAP8, a strand of pseudogene-derived lncRNA that has been shown to be associated with the occurrence and development of several kinds of tumors, as a representative lncRNA for further verification. Little knowledge is currently available on the role of DUXAP8 in endometrial cancer. Therefore, we assessed the effect of DUXAP8 on UCEC cells proliferation and colony formation *in vitro*. DUXAP8-shRNA/GFP lenti-virus (*versus* control virus) was used to knock down DUXAP8 in human UCEC cells (**Figure 10F**). Colony formation assay results showed that clonogenic survival was inhibited following the downregulation of DUXAP8 (**Figure 10G**). In addition, the growth curves detected by CCK-8 showed that DUXAP8 knockdown significantly impaired UCEC cells growth (**Figure 10H**).

## Discussion

Because of its complex etiology and genetic heterogeneity, molecular basis of UCEC is still largely unclear. Despite many efforts have been made to elucidate the pathogenesis of UCEC and to identify prognostic biomarkers, prognosis of the disease remains unfavorable, especially for advanced UCEC. Therefore, the aim of the current study was to investigate potential key molecules related to the occurrence, progression, and prognosis of UCEC, and to improve our understanding of the molecular mechanisms underlying UCEC pathogenesis.

Firstly, 2569 DEmRNA strands and 1457 DElncRNA strands were identified from 23 paired UCEC tissues and adjacent non-cancerous tissues. The co-expression network of all DEGs was then constructed to screen for modules with significant prognostic value by using the WGCNA method. Genes with similar expression patterns could be clustered into a co-expression module (34). Total of 8 co-expression modules were identified in the current study, each of which may reveal distinct regulatory mechanisms. The Blue and Brown modules were shown to be significantly associated with the neoplasm histologic grade of UCEC. Loss of differentiation is a common event in the development of a variety of human tumors, and histologic grade has been shown to be an important prognostic indicator and may influence treatment response (35). Hence, functional enrichment analyses were then performed to investigate the mechanisms of the identified modular genes in tumor differentiation. It is worth noting that all of the top 5 biological processes were relevant to mitosis (‘Mitotic nuclear division’, ‘Nuclear division’, ‘Chromosome segregation’, ‘Microtubule cytoskeleton organization involved in mitosis’ and ‘Nuclear chromosome segregation’), suggesting that mitosis exerts an important role in tumor differentiation. Consistent with the GO analysis results, KEGG pathway analysis showed that DEGs in the Blue and Brown modules were highly enriched in ‘Cell cycle’. Thereby, biological processes and pathways related to mitosis and cell cycle might play crucial roles in the progression of UCEC,

The highly connected genes of the Blue and Brown modules were further investigated as potential key factors related to the pathogenesis of UCEC. Based on MM across modules and GS for tumor differentiation, 58 genes were identified that were closely related to neoplasm histologic grade. Meanwhile, a PPI network for all genes in the two modules was constructed and 20 genes were selected as key nodes of UCEC. A total of 10 genes identified by both WGCNA and PPI analyses were designated as potential hub genes for further validation and analysis. Among the 10 candidates, 6 genes (AURKA, BUB1, CDCA8, DLGAP5, KIF2C and TPX2) were significantly negatively related to the 5-year OS of patients with UCEC, with higher expression levels in poorly differentiated UCEC tissues compared to well-differentiated UCEC tissues. All of them were reported to play pivotal roles in the development of several types of tumors (36–41). It was shown by GSEA analysis that five common gene set tightly associated with cell cycle and proliferation were enriched in the highly expressed samples, indicating vital roles of the 6 genes in the progression of UCEC. In addition, to elucidate the molecular mechanism of the identified modular lncRNA, the lncRNA-miRNA-mRNA ceRNA and lncRNA-TF-mRNA networks were analyzed and 3 lncRNA strands with prognostic and diagnostic predictive potential were finally identified, including AC015849.16, DUXAP8 and DGCR5.

Next, we clarified the expression patterns of the hub mRNAs and lncRNAs in endometrial cancer cell lines. Consistent with the results obtained for the TCGA UCEC cohort, all of these hub genes presented higher expression in KLE (histological grade 3; G3) than in Ishikawa cells (histological grade 1; G1). *In vitro* experiment results further confirmed the prognostic and diagnostic predictive potential of these hub genes. Meanwhile, given the higher degree and smaller *P* value (*P* = 0.00067), Aurora kinase A (AURKA), a member of the Aurora family of serine/threonine protein kinases, was selected as a representative hub mRNA for further verification. AURKA has been shown to exert crucial roles during cell cycle progression and mitosis. It is most expressed during G2/M phase of the cell cycle and regulates the activation of Polo Like Kinase 1 (PLK1), a crucial step for checkpoint recovery. AURKA has also been implicated in the regulation of chromosome alignment, mitotic entry, and spindle formation. Indeed, several studies have shown that AURKA is frequently upregulated in several malignancies and in some cases is related to poorer prognosis (42, 43).

Jian et al. inhibited AURKA in UCEC Ishikawa cells using small interfering RNA (siRNA) and demonstrated that AURKA silencing significantly impaired cell viability and enhanced cellular apoptosis (44). In this study, we evaluated the biological impact of suppressing AURKA with Alisertib (MLN8237), an oral and highly selective AURKA inhibitor in UCEC cells *in vitro* (45). The results demonstrated that Alisertib could significantly limit UCEC cells proliferation and colony formation. In addition, Alisertib effectively induced G2/M phase arrest and enhanced cellular apoptosis. Given that AURKA is expressed and activated only during mitosis, AURKA inhibitors likely target tumor cells relatively specifically with less damage to normal cells. Collectively, the findings suggest that AURKA is a promising therapeutic target and Alisertib represents a potential therapeutic strategy for the treatment of UCEC.

Furthermore, double homeobox A pseudogene 8 (DUXAP8), a strand of pseudogene-derived lncRNA, was selected as a representative lncRNA for functional verification. It has been shown to be associated with the pathogenesis of several kinds of tumors, such as hepatocellular carcinoma (HCC), pancreatic cancer, ovarian cancer, colorectal cancer and non-small-cell lung cancer. The direct binding of DUXAP8 to histone methyltransferase (enhancer of zeste homolog2, EZH2) and histone demethylase (Lysine Demethylase 1A, LSD1) has been firmed, indicating that DUXAP8 exerts its tumor-promotive activity at least partly through epigenetic modification of the target genes (46, 47). So far, there is no relevant report on the role of DUXAP8 in endometrial cancer and our study confirmed that knockdown of DUXAP8 significantly impaired UCEC cells growth and clonogenic ability, suggesting DUXAP8 may therefore serve as a prognostic predictor for patients with UCEC and a promising therapeutic target for UCEC treatment.

## Conclusions

Detailed understanding of the related mechanisms of UCEC pathogenesis and multidimensional interventions of the hallmarks of UCEC progression could be beneficial and warrant further exploration. Overall, the current study provides a thorough approach for the discovery of potential biomarkers in UCEC development and prognosis, extending our understanding of the molecular mechanisms underlying UCEC differentiation and progression. We identified 6 DEmRNA strands and 3 DElncRNA strands with prognostic and diagnostic predictive potential, and we experimentally validated their expression patterns in UCEC cells. Moreover, AURKA and DUXAP8 were selected as representative hub genes for functional verification, offering UCEC with potential therapeutic targets. Druggable approach with low toxicity based on manipulation of the main processes that are disturbed in UCEC, like mitosis, shows to be promising for the development of therapy for UCEC patients, and more clinical trials are needed to confirm the safety and efficacy.

## Data Availability Statement

The datasets analyzed for this study can be found in TCGA public repository (https://portal.gdc.cancer.gov/). The original contributions presented in the study are included in the article/**Supplementary Material**. Further inquiries can be directed to the corresponding authors upon reasonable request.

## Author Contributions

YY, ZC, DL, and WZ were involved in designing the study and preparing the manuscript. YY and ZC performed most of the experiments. XC and SH analyzed the data. YY, DL, and WZ contributed to critical revision of the manuscript. The corresponding authors were responsible for all aspects of the research and ensured that issues related to the accuracy or integrity of any part of the work were investigated and resolved. All authors reviewed and approved the final version of the manuscript.

## Funding

This work was supported by the grants from National Natural Science Foundation of China (82002222, 82172774) and National Postdoctoral Science Foundation of China (2020M681400).

## Conflict of Interest

The authors declare that the research was conducted in the absence of any commercial or financial relationships that could be construed as a potential conflict of interest.

## Publisher’s Note

All claims expressed in this article are solely those of the authors and do not necessarily represent those of their affiliated organizations, or those of the publisher, the editors and the reviewers. Any product that may be evaluated in this article, or claim that may be made by its manufacturer, is not guaranteed or endorsed by the publisher.
